# Trends in Patient Characteristics and COVID-19 In-Hospital Mortality in the United States During the COVID-19 Pandemic

**DOI:** 10.1001/jamanetworkopen.2021.8828

**Published:** 2021-05-03

**Authors:** Gregory A. Roth, Sophia Emmons-Bell, Heather M. Alger, Steven M. Bradley, Sandeep R. Das, James A. de Lemos, Emmanuela Gakidou, Mitchell S. V. Elkind, Simon Hay, Jennifer L. Hall, Catherine O. Johnson, David A. Morrow, Fatima Rodriguez, Christine Rutan, Saate Shakil, Reed Sorensen, Laura Stevens, Tracy Y. Wang, Jason Walchok, Joseph Williams, Christopher Murray

**Affiliations:** 1 Division of Cardiology, Department of Medicine, University of Washington, Seattle; 2 American Heart Association, Dallas, Texas; 3 Minneapolis Heart Institute, Minneapolis, Minnesota; 4 Minneapolis Heart Institute Foundation, Minneapolis, Minnesota; 5 Associate Editor, *JAMA Network Open*; 6 Cardiology Division, Department of Internal Medicine, University of Texas Southwestern Medical Center, Dallas; 7 Parkland Health and Hospital System, Dallas, Texas; 8 Department of Neurology, Vagelos College of Physicians and Surgeons, New York, New York; 9 Department of Epidemiology, Mailman School of Public Health, Columbia University, New York, New York; 10 University of Minnesota, Minneapolis; 11 Cardiovascular Division, Brigham and Women’s Hospital, Boston, Massachusetts; 12 Cardiovascular Institute, Division of Cardiovascular Medicine, Stanford University, Stanford, California; 13 University of Colorado Anschutz Medical Campus, Aurora; 14 Duke Clinical Research Institute, Durham, North Carolina

## Abstract

**Importance:**

In-hospital mortality rates from COVID-19 are high but appear to be decreasing for selected locations in the United States. It is not known whether this is because of changes in the characteristics of patients being admitted.

**Objective:**

To describe changing in-hospital mortality rates over time after accounting for individual patient characteristics.

**Design, Setting, and Participants:**

This was a retrospective cohort study of 20 736 adults with a diagnosis of COVID-19 who were included in the US American Heart Association COVID-19 Cardiovascular Disease Registry and admitted to 107 acute care hospitals in 31 states from March through November 2020. A multiple mixed-effects logistic regression was then used to estimate the odds of in-hospital death adjusted for patient age, sex, body mass index, and medical history as well as vital signs, use of supplemental oxygen, presence of pulmonary infiltrates at admission, and hospital site.

**Main Outcomes and Measures:**

In-hospital death adjusted for exposures for 4 periods in 2020.

**Results:**

The registry included 20 736 patients hospitalized with COVID-19 from March through November 2020 (9524 women [45.9%]; mean [SD] age, 61.2 [17.9] years); 3271 patients (15.8%) died in the hospital. Mortality rates were 19.1% in March and April, 11.9% in May and June, 11.0% in July and August, and 10.8% in September through November. Compared with March and April, the adjusted odds ratios for in-hospital death were significantly lower in May and June (odds ratio, 0.66; 95% CI, 0.58-0.76; *P* < .001), July and August (odds ratio, 0.58; 95% CI, 0.49-0.69; *P* < .001), and September through November (odds ratio, 0.59; 95% CI, 0.47-0.73).

**Conclusions and Relevance:**

In this cohort study, high rates of in-hospital COVID-19 mortality among registry patients in March and April 2020 decreased by more than one-third by June and remained near that rate through November. This difference in mortality rates between the months of March and April and later months persisted even after adjusting for age, sex, medical history, and COVID-19 disease severity and did not appear to be associated with changes in the characteristics of patients being admitted.

## Key Points

**Question:**

What factors are associated with observed trends in the in-hospital mortality rates in the United States during the first 9 months of the COVID-19 pandemic?

**Findings:**

In this cohort study of 20 736 patients, in-hospital mortality rates decreased in the US between March and November 2020, even after accounting for the changing mix in patient age, sex, comorbidities, and disease severity at the time of admission. Hospital and intensive care unit length of stay and use of mechanical ventilation decreased over time, whereas the use of glucocorticoids and remdesivir increased.

**Meaning:**

Changes in age, sex, comorbidities, and disease severity among patients with COVID-19 do not fully explain the decrease in the in-hospital mortality rates observed during the first 9 months of the COVID-19 pandemic.

## Teaser

This cohort study of patients included in the US American Heart Association COVID-19 Cardiovascular Disease Registry evaluates whether any changes in the in-hospital COVID-19 mortality rates during the first 9 months of the pandemic are associated with individual characteristics of patients with COVID-19.

## Introduction

Understanding the course of COVID-19 among hospitalized patients is important for clinicians seeking to improve health outcomes in this high-risk population.^1,2^ Estimates of in-hospital mortality rates among patients with COVID-19 are particularly important for tracking the quality and effectiveness of hospital care.^3^

Surveillance has proven particularly challenging in the United States, where no single registry tracks all COVID-19 cases. The American Heart Association’s (AHA) COVID-19 Cardiovascular Disease (CVD) Registry powered by Get With The Guidelines was deployed in April 2020 to address this gap and as a means of tracking hospital-based outcomes and improving national surveillance of all patients with COVID-19 admitted to a hospital.^4^

The objective of this analysis of the AHA COVID-19 CVD Registry was to quantify changes in in-hospital mortality rates during the first 9 months of the pandemic and to understand if any observed changes were associated with differences over time in the characteristics of patients presenting to the hospital. Such changes in patient characteristics are suggested by the course of the pandemic, which had devastating impacts on older populations in nursing homes in the first months of the pandemic and more widespread effects in subsequent months. In particular, we sought to examine these temporal trends in in-hospital mortality rates after accounting for differences in age, sex, preexisting health status, and disease severity at admission. We hypothesized that in-hospital fatality has decreased in the US since March, and that these decreases are not simply associated with changes in the profile of admitted patients.

## Methods

### Data Source and Participants

This study was a retrospective analysis of the AHA COVID-19 CVD Registry, undertaken as part of the Global Burden of Disease Study. Details of the registry have been previously described.^4,5^ Patient information was extracted from medical records by trained abstractors using standardized definitions and entered in a database. Hospitals voluntarily joined the registry throughout the period of analysis. Each participating hospital was instructed to abstract data for all patients 18 years or older hospitalized with a confirmed diagnosis of SARS-CoV-2. Cases were confirmed by clinical diagnosis using hospital criteria, reverse transcriptase–polymerase chain reaction analysis, or an IgM antibody test. There were 355 patients with a second admission, which were excluded from the analysis because these repeat admissions were likely to differ substantially in nature from index admissions. The research team did not have access to patient identifiers and performed the analysis using a limited, deidentified version of the database in a secure workspace of the AHA Precision Medicine Platform.^6^ IQVIA managed the data collection platform, and the Duke Clinical Research Institute served as the coordinating center. We followed the Strengthening the Reporting of Observational Studies in Epidemiology (STROBE) reporting guideline. The University of Washington Institutional Review Board Committee approved the study and waived the need for obtaining informed consent because data were collected as part of routine quality-improvement activities and no direct patient interactions or postdischarge follow-up occurred. No one received compensation or was offered any incentive for participating in this study.

### Patient Characteristics

Patient characteristics were considered and included in statistical analyses based on their availability in the registry, clinical rationale, and previously published studies on COVID-19. Medical history was entered as a binary indicator in the database by hospital personnel based on review of the medical record. The following characteristics were considered: age, sex, and medical history, including hypertension, smoking, diabetes, prior coronary revascularization, heart failure, cancer, chronic kidney disease, and pulmonary disease (including chronic obstructive pulmonary disease, asthma, or interstitial lung disease). Additional data collected from the time of admission, when available, included body mass index (BMI; calculated as weight in kilograms divided by height in meters squared), oxygen saturation percent, respiratory rate, heart rate, systolic blood pressure, creatinine concentrations, use of supplemental oxygen at admission, and the presence of pulmonary infiltrates on initial chest radiographic images or chest computed tomographic images at admission.

### Outcome Measure

The outcome measure of interest was in-hospital death. Deaths were assigned to the date of admission. We adjusted only for patient characteristics known at the time of admission to evaluate our hypothesis that decreases in mortality rates were not due to a change in the profile of admitted patients. Differences in in-hospital based interventions (only a subset of which were recorded in this registry) remain a possible explanation for this decrease.

### Statistical Analysis

Statistical analysis was performed using R, version 3.6.0 (R Foundation). Measures of central tendency were calculated, distributions were visualized, and univariable associations were estimated. Continuous variables were binned into clinically relevant categories. Dummy variables were created to indicate hospital admissions that occurred during 4 periods in 2020: March and April; May and June; July and August; or September through November. Prior to conducting a regression model, multiple imputation was performed using the mice package in R to account for missing values (eTable 1 in the Supplement).^7^ A multiple mixed-effects logistic regression was then used to estimate the odds of in-hospital death adjusted for patient age, sex, BMI, and medical history as well as vital signs, use of supplemental oxygen, and presence of pulmonary infiltrates at admission. β-Coefficients were exponentiated and then interpreted as an odds ratio (OR) and 95% CI. Independent variables consisted of the covariates described above and an indicator variable for time period. Random intercepts were placed on hospital of admission to account for within- and across-hospital variability. Because age is known to be the dominant risk factor of mortality, we also examined adjusted in-hospital mortality rates separately for the following age categories: 41 to 50, 51 to 60, 61 to 70, 71 to 80, and older than 80 years. Values of *P* < .05 were considered statistically significant.

## Results

### Study Cohort and Patient Characteristics

Our analysis included 20 736 patients admitted between March and November 2020 at 107 hospitals in 31 states, with 11 901 patients admitted in March or April, 4116 patients in May or June, 2709 in July or August, and 2010 patients in September through November (eFigure in the Supplement). Patient characteristics are given in the Table. The mean (SD) age was 61.2 (17.9) years, and 9524 (45.9%) were women. Hypertension was the most common preexisting condition recorded, present in more than half of patients, followed by diabetes in just over a third of patients. Pulmonary disease (including chronic obstructive pulmonary disease) was present in almost one-fifth of patients. Cancer was present in 2560 patients (12.3%), chronic kidney disease in 2628 patients (12.7%), and heart failure in 2343 patients (11.3%). At the time of admission to the hospital, the mean (SD) BMI was in the obese range (ie, 30.8 [8.5]), and approximately a quarter of patients were receiving supplemental oxygen. The mean (SD) creatinine concentration (1.7 [5.4] mg/dL) was above reference ranges (to convert to micromoles per liter, multiply by 88.4), and two-thirds of patients had interstitial infiltrates on chest radiographic or computed tomographic images. Almost one-third of patients were admitted to an intensive care unit, with 1 in 5 patients being placed on mechanical ventilation. Azithromycin was administered to nearly half the patients; glucocorticoids, to more than one-third of patients.

**Table.** Demographic and Clinical Characteristics of Patients Admitted to the Hospital With COVID-19 for 4 Periods in 2020

Characteristic	Patients, No. (%)				
	March and April (n = 11 901)	May and June (n = 4116)	July and August (n = 2709)	September-November (n = 2010)	Total (N = 20 736)
Men	6709 (56.4)	2080 (50.5)	1386 (51.2)	1037 (51.6)	11 212 (54.1)
Women	5192 (43.6)	2036 (49.5)	1323 (48.8)	973 (48.4)	9524 (45.9)
Age, mean (SD), y	62.1 (17.3)	59.6 (18.9)	59.2 (18.4)	61.4 (18.1)	61.2 (17.9)
Survival status					
In-hospital death	2268 (19.1)	488 (11.9)	298 (11.0)	217 (10.8)	3271 (15.8)
Medical history					
CABG or PCI	749 (6.3)	247 (6.0)	173 (6.4)	193 (9.6)	1362 (6.6)
Cancer	1603 (13.5)	408 (9.9)	284 (10.5)	265 (13.2)	2560 (12.3)
Cerebrovascular disease	1522 (12.8)	486 (11.8)	255 (9.4)	198 (9.9)	2461 (11.9)
Chronic kidney disease	1485 (12.5)	556 (13.5)	315 (11.6)	272 (13.5)	2628 (12.7)
Diabetes	4043 (34.0)	1523 (37.0)	997 (36.8)	698 (34.7)	7261 (35.0)
Heart failure	1226 (10.3)	514 (12.5)	331 (12.2)	272 (13.5)	2343 (11.3)
Hypertension	6865 (57.7)	2382 (57.9)	1654 (61.1)	1215 (60.4)	12 116 (58.4)
Pulmonary disease	2144 (18.0)	705 (17.1)	446 (16.5)	507 (25.2)	3802 (18.3)
Smoking	713 (6.0)	283 (6.9)	180 (6.6)	156 (7.8)	1332 (6.4)
Admission characteristic					
BMI, mean (SD)	30.4 (8.1)	30.9 (8.6)	31.8 (9.2)	31.6 (9.0)	30.8 (8.5)
O_2_ saturation, mean (SD), %	93.4 (6.9)	94.3 (6.5)	93.9 (7.0)	94 (6.2)	93.7 (6.8)
Supplemental O_2_	2733 (23.0)	1256 (30.5)	885 (32.7)	722 (35.9)	5596 (27.0)
Interstitial infiltrates	8416 (70.7)	2528 (61.4)	1549 (57.2)	1223 (60.8)	13 716 (66.1)
Respiratory rate, mean (SD) breaths/min	21.5 (6.7)	21.8 (6.7)	21.3 (6.5)	21.3 (6.5)	21.5 (6.6)
Heart rate, mean (SD) beats/min	95 (19.6)	93.5 (19.9)	91.8 (19.7)	91 (19.9)	93.9 (19.8)
Systolic BP, mean, (SD), mm Hg	130.9 (24.0)	131.1 (24.0)	131.2 (23.9)	131.6 (23.4)	131 (23.9)
Creatinine, mean (SD), mg/dL	1.7 (5.4)	1.8 (6.7)	1.7 (4.5)	1.5 (3.6)	1.7 (5.4)
Hospitalization characteristic					
Length of stay, mean (SD), d	10.7 (12.1)	9.6 (10.5)	9.3 (9.9)	7.5 (6.8)	10 (11.1)
ICU admission	3641 (30.6)	1143 (27.8)	915 (33.8)	686 (34.1)	6385 (30.8)
Length of stay in ICU, mean (SD), d	13.9 (14.1)	10.7 (11.7)	9.6 (11.1)	6.6 (7.0)	11.9 (12.9)
Placed on mechanical ventilation	2768 (23.3)	593 (14.4)	426 (15.7)	279 (13.9)	4066 (19.6)
Days on ventilator, mean (SD)	12.3 (12.9)	11.5 (12.2)	11.8 (13.1)	7.1 (7.1)	11.8 (12.5)
Treated with					
Glucocorticoids	2632 (22.1)	1417 (34.4)	1694 (62.5)	1374 (68.4)	7117 (34.3)
Remdesivir	448 (3.8)	873 (21.2)	886 (32.7)	931 (46.3)	3138 (15.1)
Azithromycin	6169 (51.8)	1341 (32.6)	790 (29.2)	439 (21.8)	8739 (42.1)
Immunoglobulins	78 (0.7)	29 (0.7)	29 (1.1)	6 (0.3)	142 (0.7)

Abbreviations: BMI, body mass index (calculated as weight in kilograms divided by height in meters squared); BP, blood pressure; CABG, coronary artery bypass grafting; ICU, intensive care unit; O_2_, oxygen; PCI, percutaneous coronary intervention.

SI conversion factor: To convert creatinine to micromoles per liter, multiply by 88.4.

### Trends in Hospital Presentation and Care

The mean (SD) age decreased from 62.1 (17.3) years in March and April to 61.4 (18.1) years in September through November. The proportion of women increased slightly over time, with 5192 women of 11 901 patients (43.6%) admitted in March and April, and 973 women of 2010 patients (48.4%) in September through November. The mean BMI at admission also increased a small amount. The percentage of patients receiving supplemental oxygen on admission increased from 2733 of 11 901 patients (23.0%) in March and April to 722 of 2010 patients (35.9%) in September through November, despite the presence of interstitial infiltrates on admission decreasing from 8416 of 11 901 patients (70.7%) to 1223 of 2010 patients (60.8%) during the same periods. The mean (SD) hospital length of stay decreased from 10.7 (12.1) days to 7.5 (6.8) days, and use of mechanical ventilation decreased substantially from 2768 of 11 901 patients (23.3%) to 279 of 2010 patients (13.9%) during the same periods (eTable 2 in the Supplement). Use of glucocorticoids and remdesivir increased substantially.

### Outcome

Of 20 736 patients, there were 3271 in-hospital deaths recorded (15.8% in-hospital mortality). There were 2268 deaths among 11 901 patients (19.1% in-hospital mortality) admitted in March and April, 488 deaths among 4116 patients (11.9% in-hospital mortality) admitted in May and June, 298 deaths among 2709 patients (11.0% in-hospital mortality) admitted in July and August, and 217 among 2010 patients (10.8% in-hospital mortality) admitted in September through November. In-hospital mortality rates are given separately for each month in eTable 4 in the Supplement. Compared with March and April, the adjusted odds for in-hospital death were significantly lower in May and June (OR, 0.66; 95% CI, 0.58-0.76; *P* < .001), July and August (OR, 0.58; 95% CI, 0.49-0.69; *P* < .001), and September through November (OR, 0.59; 95% CI, 0.47-0.73; *P* < .001) (Figure). In this multivariate model, age was most strongly associated with death. When examined separately in analyses stratified by 10-year age groups, and in comparison with March and April, adjusted in-hospital mortality rates were significantly lower in May and June, July and August, and September through November for patients in age groups 51 to 60, 61 to 70, 71 to 80, and older than 80 years (Figure). Male sex, BMI greater than 45, cancer, cerebrovascular disease, diabetes, and heart failure were also independently associated with in-hospital death (eTable 3 in the Supplement).

**Figure.**
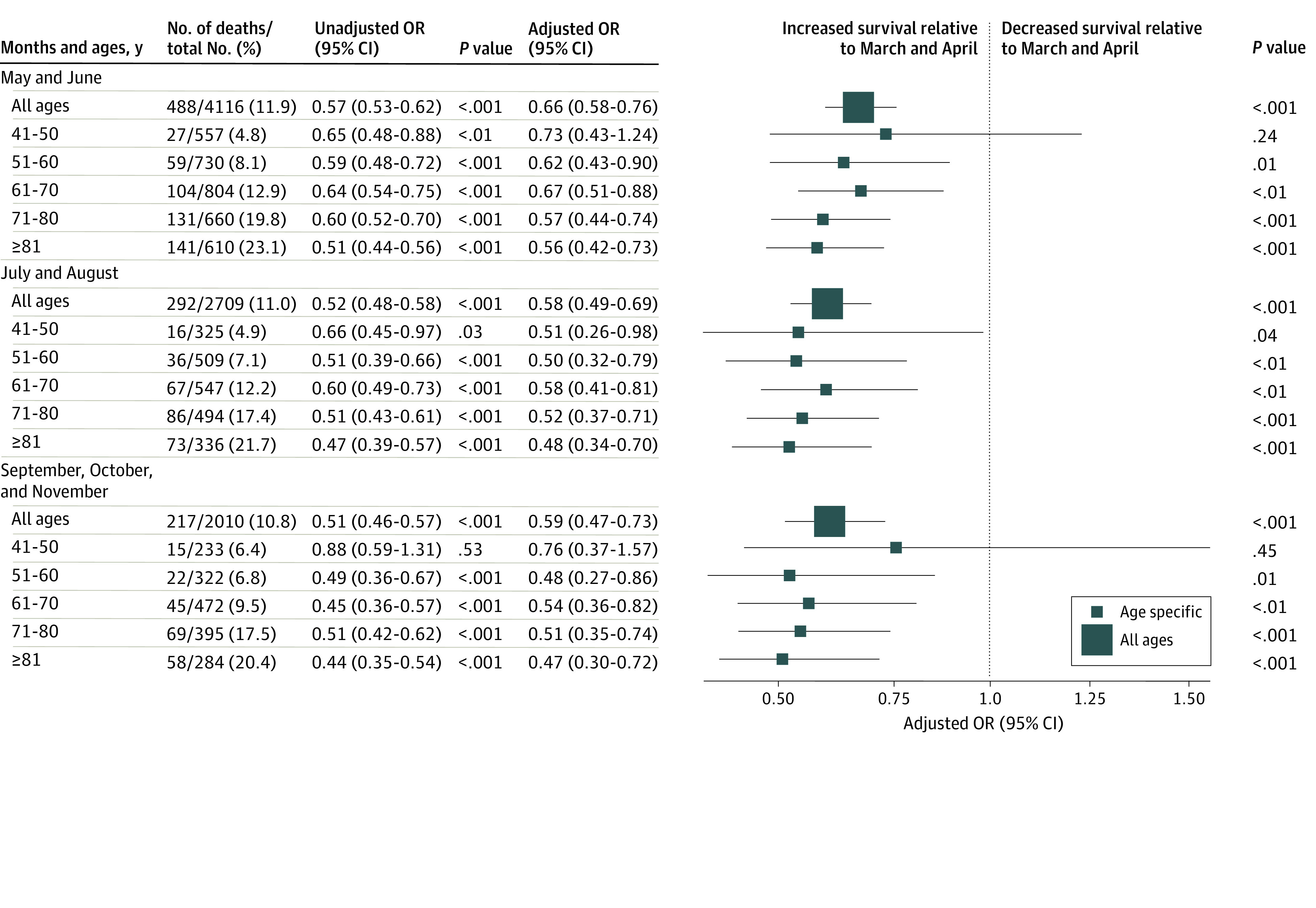
Unadjusted and Adjusted Odds of Inpatient Death in May and June, July and August, and September Through November Compared With March and April, 2020 Odds ratios (ORs) calculated as the exponentiated effect size of each logistic regression. Unadjusted ORs represent logistic regression of inpatient death against month, stratified by age. Adjusted ORs represent logistic regression of inpatient death against month and characteristics known at admission (sex, heart rate, respiratory rate, systolic blood pressure, creatinine concentration, oxygen saturation, presence of interstitial infiltrates, and use of supplemental oxygen, and history of cancer, cerebrovascular disease, chronic kidney disease, heart failure, diabetes, hypertension, coronary artery bypass grafting or percutaneous coronary intervention, pulmonary disease, and smoking), stratified by age, with a random effect on hospital identification.

## Discussion

Changes in the in-hospital mortality rates during the course of the pandemic have been difficult to interpret in the United States owing to waves of SARS-CoV-2 infection occurring in different states in different periods and among populations with varying patterns of age and comorbid conditions.^3^ In this analysis, we found the greatest decrease in the in-hospital mortality rates to have occurred between March and May 2020. There were only small changes in the characteristics of patients admitted with COVID-19 for a 9-month period from March through November 2020, with a decrease in age, increase in proportion of women, and increase in BMI over time. The in-hospital mortality rate was lower among patients admitted after March and April, even after accounting for these characteristics as well as medical history and disease severity at the time of admission, including vital signs and presence of pulmonary infiltrates on chest imaging. Our analysis suggests that these improvements in mortality rates may be sustained in the face of the rapidly increasing hospital admission rates currently being observed in the United States.

Our analysis suggests several hypotheses that could explain decreasing in-hospital mortality rates. Extremely high hospital census or rapid implementation of new isolation and personal protection procedures may have adversely impacted patient outcomes in locations with very high rates of COVID-19 in March and April. This hypothesis is consistent with our observation of in-hospital mortality rates decreasing most rapidly between the months of March and April and the months of May and June as hospital census decreased and health care workers became more familiar with new procedures. The use of supplemental oxygen at admission increased and use of mechanical ventilation decreased between these 2 time periods even though rates of intensive care unit admissions changed only slightly. Increased use of noninvasive ventilation, high flow nasal oxygen, and prone positioning could explain these trends in respiratory care and are associated with improved outcomes.^8,9,10^ The use of glucocorticoids and remdesivir increased substantially in registry patients as data on their efficacy accumulated and the US Food and Drug Administration announced a remdesivir emergency use authorization on May 1, 2020.^11,12^ The absolute mortality rate decrease to be expected from the use of remdesivir remains unclear in the face of mixed study results, and there is no evidence for the efficacy of azithromycin in treating COVID-19.^13,14,15^

Our analysis substantially expands the timeline for analysis of in-hospital mortality rates 5 months beyond what has been previously reported. In an analysis using a large sample of electronic claims data, Asch et al^16^ showed that in-hospital mortality rates had been improving in the United States through the end of June 2020. Decreasing in-hospital mortality rates have also been reported previously for individual health systems. A 3-hospital system in New York City reported a relative decrease of 70% in adjusted mortality rates (from 25.6% in March to 7.6% in August), which is a larger decrease than the 43% relative decrease observed in the 107 hospitals in our analysis. Improvements in survival rates among critical care patients has also been reported for intensive care units in England, a trend that persisted after adjustment for age, sex, and comorbidities.^17^ An 8-hospital system in Houston reported decreases in unadjusted in-hospital mortality rates from 12.1% during March to mid-May to 5.1% from mid-May until July.^18^ Our results using a large national sample add additional detail previously unavailable, including vital signs at time of admission, the frequency of pulmonary infiltrates on chest imaging, and use of inpatient therapies such as glucocorticoids.

Female sex was associated with survival in our analysis. Some studies have shown enhanced immunity against SARS-CoV-2 in women compared with men.^19^ We also found increased risk among patients with a BMI greater than 45.^20^ Our findings persisted despite adjustment for vital signs and oxygen saturation at admission, consistent with a study of 4 hospitals in New York City that also adjusted for vital signs and found increased risk when the BMI exceeded 40.^21^ Further investigation is needed to understand what mechanisms explain associations between patient characteristics and COVID-19 outcomes.

### Limitations

Our analysis was retrospective and relied on data extracted at each hospital from clinical medical records. Although these data are among the most detailed available in the United States for patients hospitalized with COVID-19, bias due to confounding from unobserved or unrecorded characteristics could remain in our estimation of associations, and we cannot confirm causality. Sample size in each of the 4 periods of observation and the composition of hospitals in the database varied due to the course of the pandemic in the US and to voluntary hospital enrollment in the registry system. To account for these factors, we chose the individual patient as the unit of analysis, with a single model used across all periods that placed a random intercept on hospital of admission to account for within and across-hospital variability. The registry did not include data on noninvasive ventilation, delivery of high-flow oxygen, or prone positioning of patients; therefore, not all interventions that may explain changes in mortality rates could be explored in this analysis. Finally, mortality rates in November may be biased lower due to deaths that occurred after the last date of available registry data on November 28, 2020.

## Conclusions

The high rates of in-hospital COVID-19 mortality among US registry patients in March and April 2020 fell by 38% by May and June with a modest further decrease by November. This difference in mortality rates between the months of March and April and these later months persisted even after adjusting for age, sex, medical history, and COVID-19 disease severity. There is an urgent need to identify, share, and implement best practices for hospital care to prevent in-hospital mortality rates from increasing again.

## Notes

Supplement. **eFigure.** Study Flowchart of Patients With COVID-19 Enrolled in AHA GWTG Registry **eTable 1.** Missingness by Variable **eTable 2.** Length of Stay by Month and Age Group **eTable 3.** Univariate and Multivariate Association With In-Hospital Death **eTable 4.** Counts of Inpatients and Deaths, and In-Hospital Mortality Rate, by Month
