# Supplementary material for: Trends in Patient Characteristics and COVID-19 In-Hospital Mortality in the United States During the COVID-19 Pandemic
Source: JAMA Netw Open. 2021 May 3;4(5):e218828. doi: 10.1001/jamanetworkopen.2021.8828 (PMC8094014; doi:10.1001/jamanetworkopen.2021.8828)
Supplement: Supplement. — eFigure. Study Flowchart of Patients With COVID-19 Enrolled in AHA GWTG Registry eTable 1. Missingness by Variable eTable 2. Length of Stay by Month and Age Group eTable 3. Univariate and Multivariate Association With In-Hospital Death eTable 4. Counts of Inpatients and Deaths, and In-Hospital Mortality Rate, by Month [file jamanetwopen-e218828-s001.pdf]

Supplementary Online Content

Roth GA, Emmons-Bell S, Alger HM, et al. Trends in patient characteristics and COVID-19 in-hospital mortality in the United States during the COVID-19 pandemic. *JAMA Netw Open*. 2021;4(5):e218828. doi:10.1001/jamanetworkopen.2021.8828

eFigure. Study Flowchart of Patients With COVID-19 Enrolled in AHA GWTG Registry
eTable 1. Missingness by Variable
eTable 2. Length of Stay by Month and Age Group
eTable 3. Univariate and Multivariate Association With In-Hospital Death
eTable 4. Counts of Inpatients and Deaths, and In-Hospital Mortality Rate, by Month

This supplementary material has been provided by the authors to give readers additional information about their work.

© 2021 Roth GA et al. *JAMA Network Open*.

eFigure. Study Flowchart of Patients With COVID-19 Enrolled in AHA GWTG Registry

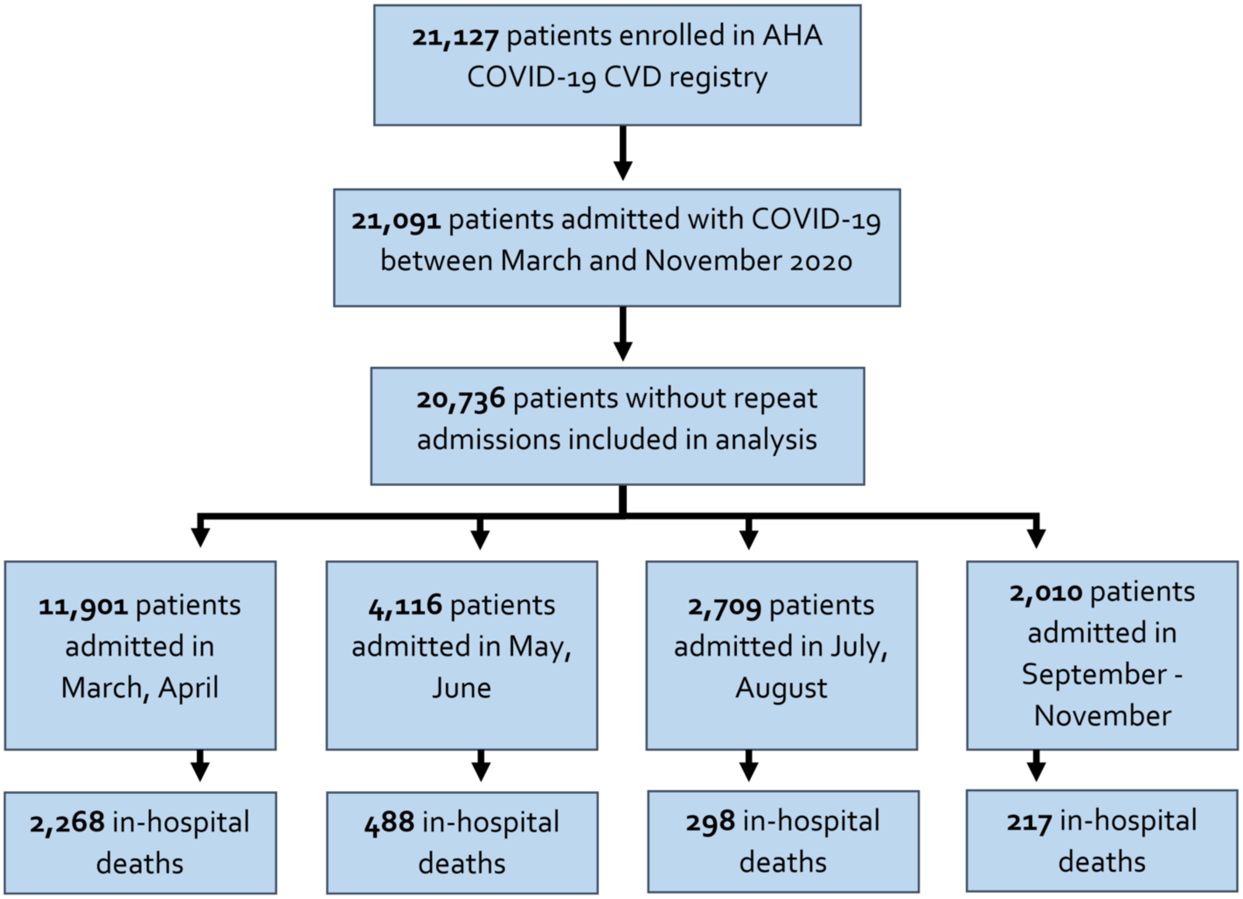

© 2021 Roth GA et al. *JAMA Network Open*.

eTable 1. Missingness by Variable

	**March, April** (n=11,901)	**May, June** (n=4,116)	**July, August** (n=2,709)	**September, November** (n=2,010)	**Total** (n=20,736)
Women	0 (0%)	0 (0%)	0 (0%)	0 (0%)	0 (0%)
Age	0 (0%)	0 (0%)	0 (0%)	0 (0%)	0 (0%)
**Survival status**					
In-hospital death	0 (0%)	0 (0%)	0 (0%)	0 (0%)	0 (0%)
**Past medical history**					
CABG or PCI	0 (0%)	0 (0%)	0 (0%)	0 (0%)	0 (0%)
Cancer	0 (0%)	0 (0%)	0 (0%)	0 (0%)	0 (0%)
Cerebrovascular disease	0 (0%)	0 (0%)	0 (0%)	0 (0%)	0 (0%)
Chronic kidney disease	0 (0%)	0 (0%)	0 (0%)	0 (0%)	0 (0%)
Diabetes	0 (0%)	0 (0%)	0 (0%)	0 (0%)	0 (0%)
Heart failure	0 (0%)	0 (0%)	0 (0%)	0 (0%)	0 (0%)
Hypertension	0 (0%)	0 (0%)	0 (0%)	0 (0%)	0 (0%)

© 2021 Roth GA et al. *JAMA Network Open*.

Pulmonary disease	0 (0%)	0 (0%)	0 (0%)	0 (0%)	0 (0%)
Smoking	0 (0%)	0 (0%)	0 (0%)	0 (0%)	0 (0%)
**Admission Characteristics**					
BMI, kg/m^2	1,854 (15.6%)	326 (7.9%)	120 (4.4%)	64 (3.2%)	7,092 (11.4%)
O2 saturation, %	1,239 (10.4%)	182 (4.4%)	37 (1.4%)	16 (0.8%)	4,422 (7.1%)
Supplemental O2	1,239 (10.4%)	184 (4.5%)	42 (1.6%)	17 (0.8%)	4,446 (7.1%)
Interstitial infiltrates	1,028 (8.6%)	376 (9.1%)	453 (16.7%)	212 (10.5%)	6,207 (10%)
Respiratory rate	770 (6.5%)	70 (1.7%)	32 (1.2%)	25 (1.2%)	2,691 (4.3%)
Heart rate	698 (5.9%)	62 (1.5%)	28 (1%)	8 (0.4%)	2,388 (3.8%)
Systolic BP, mmHg	724 (6.1%)	134 (3.3%)	170 (6.3%)	44 (2.2%)	3,216 (5.2%)
Creatinine, mg/dL	868 (7.3%)	135 (3.3%)	75 (2.8%)	55 (2.7%)	3,399 (5.5%)

Values represent number, and percent, of admissions with missing values for each covariate.

© 2021 Roth GA et al. *JAMA Network Open*.

eTable 2. Length of Stay by Month and Age Group

Length of Stay (days)	March - April	May - June	July - August	September - November	Total
18-50, mean (SD)	8.7 (10.4)	7.6 (10.1)	7.3 (8.4)	6.0 (6.1)	8.0 (9.8)
51-60, mean (SD)	11.2 (12.9)	10.1 (10.8)	9.5 (11.0)	7.6 (6.9)	10.5 (11.9)
61-70, mean (SD)	12.4 (13.8)	11.1 (11.3)	11.0 (11.3)	8.3 (7.7)	11.5 (12.6)
71-80, mean (SD)	11.9 (12.8)	11.2 (10.8)	10.5 (10.1)	8.3 (6.7)	11.2 (11.7)
81 plus, mean (SD)	9.5 (9.2)	9.7 (8.6)	9.2 (7.6)	7.6 (6.3)	9.4 (8.7)
All ages, total days	126,910	39,526	25,163	15,103	206,702

© 2021 Roth GA et al. *JAMA Network Open*.

eTable 3. Univariate and Multivariate Association With In-Hospital Death

	Univariate association with mortality	P-value, Univariate association with mortality	Multivariate association with mortality	P-value, multivariate association with mortality
Women	0.72 (0.66 - 0.77)	<.001	0.74 (0.68 - 0.81)	<.001
Age 18 - 25	0.16 (0.08 - 0.30)	<.001	0.26 (0.13 - 0.51)	<.001
Age 26 - 30	0.31 (0.19 - 0.50)	<.001	0.45 (0.28 - 0.75)	<.01
Age 31 - 35	0.34 (0.23 - 0.51)	<.001	0.43 (0.29 - 0.66)	<.001
Age 36 - 40	0.46 (0.33 - 0.65)	<.001	0.54 (0.37 - 0.77)	<.001
Age 41 - 45	0.44 (0.32 - 0.60)	<.001	0.44 (0.32 - 0.62)	<.001
Age 46 - 50	0.81 (0.63 – 1.03)	.09	0.86 (0.66 - 1.12)	.26
Age 51 - 55	Reference category	N/A	Reference category	N/A
Age 56 - 60	1.24 (1.01 - 1.53)	.04	1.2 (0.96 - 1.51)	.11
Age 61 - 65	1.43 (1.17 - 1.75)	<.01	1.35 (1.09 - 1.68)	<.01
Age 66 - 70	2.19 (1.81 - 2.66)	<.001	1.99 (1.61 - 2.46)	<.001
Age 71 - 75	2.82 (2.33 - 3.42)	<.001	2.71 (2.19 - 3.36)	<.001
Age 76 - 80	3.38 (2.79 - 4.10)	<.001	3.41 (2.75 - 4.23)	<.001
Age 81 - 85	3.73 (3.06 - 4.55)	<.001	4.21 (3.36 - 5.28)	<.001
Age 86 - 90	4.62 (3.76 – 5.70)	<.001	5.16 (4.06 - 6.57)	<.001
Age 91 - 95	4.95 (3.90 - 6.27)	<.001	5.65 (4.28 - 7.45)	<.001
Age 96 - 110	4.70 (3.34 – 6.62)	<.001	5.07 (3.43 - 7.49)	<.001
**Past medical history**				
CABG or PCI	1.83 (1.60 - 2.08)	<.001	1.08 (0.92 - 1.26)	.33
Cancer	1.63 (1.47 - 1.80)	<.001	1.25 (1.1 - 1.41)	<.001
Cerebrovascular disease	1.80 (1.63 – 1.99)	<.001	1.2 (1.05 - 1.37)	<.01
Chronic kidney disease	1.95 (1.77 - 2.15)	<.001	0.88 (0.77 - 1.01)	.06
Diabetes	1.46 (1.35 - 1.58)	<.001	1.1 (1 - 1.21)	.04
Heart failure	2.15 (1.94 - 2.34)	<.001	1.22 (1.07 - 1.38)	<.01
Hypertension	1.94 (1.79 - 2.10)	<.001	1.07 (0.96 - 1.19)	.24
Pulmonary disease	1.31 (1.20 - 1.44)	<.001	1.07 (0.96 - 1.2)	.22
Smoking	1.08 (0.93 - 1.25)	.32	1.11 (0.93 - 1.33)	.25
**Admission characteristic**				
BMI < 20, kg/m^2	1.03 (0.87 - 1.23)	.72	0.84 (0.69 - 1.03)	.09
BMI 20 – 24.9, kg/m^2	Reference category	N/A	Reference category	N/A

© 2021 Roth GA et al. *JAMA Network Open*.

BMI 25 – 29.9, kg/m^2	0.84 (0.76 - 0.94)	<.01	1.01 (0.89 - 1.14)	.90
BMI 30 – 34.9 , kg/m^2	0.70 (0.63 - 0.79)	<.001	1.08 (0.94 - 1.24)	.28
BMI 35 – 39.9, kg/m^2	0.70 (0.61 - 0.80)	<.001	1.18 (1 - 1.4)	.05
BMI 40 – 44.9, kg/m^2	0.67 (0.56 - 0.80)	<.001	1.18 (0.95 - 1.46)	.13
BMI >= 45, kg/m^2	0.71 (0.59 - 0.84)	<.001	1.44 (1.16 - 1.79)	<.001
O2 saturation <=70%	7.79 (6.21 – 9.76)	<.001	6.91 (5.24 - 9.11)	<.001
O2 saturation 71 - 85%	3.45 (3.01 – 3.95)	<.001	3 (2.54 - 3.53)	<.001
O2 saturation 86 - 93%	1.65 (1.51 - 1.79)	<.001	1.5 (1.36 - 1.66)	<.001
O2 saturation 94 – 100%	Reference category	N/A	Reference category	N/A
Supplemental O2, n (%)	3.20 (2.97 - 3.46)	<.001	2.82 (2.56 - 3.1)	<.001
Interstitial infiltrates	2.22 (2.00 - 2.45)	<.001	1.58 (1.41 - 1.78)	<.001
Respiratory rate <= 20	Reference category	N/A	Reference category	N/A
Respiratory rate 21 - 25	1.69 (1.54 - 1.86)	<.001	1.37 (1.23 - 1.53)	<.001
Respiratory rate 26 - 30	2.12 (1.89 - 2.38)	<.001	1.59 (1.39 - 1.82)	<.001
Respiratory rate >30	3.44 (3.05 – 3.88)	<.001	2.08 (1.78 - 2.42)	<.001
Heart rate <=100	Reference category	N/A	Reference category	N/A
Heart rate 101 - 130	1.09 (1.00 - 1.18)	.04	1.3 (1.18 - 1.43)	<.001
Heart rate >130	1.84 (1.55 - 2.20)	<.001	1.9 (1.52 - 2.36)	<.001
Systolic BP <= 110 mmHg	1.61 (1.42 - 1.81)	<.001	1.26 (1.09 - 1.45)	<.01
Systolic BP 111 – 120 mmHg	Reference category	N/A	Reference category	N/A
Systolic BP 121 – 150 mmHg	0.91 (0.82 – 1.02)	.12	0.9 (0.79 - 1.03)	.11
Systolic BP > 150 mmHg	0.97 (0.86 - 1.11)	.70	0.82 (0.71 - 0.96)	.01
Creatinine <=1.5 mg/dL	Reference category	N/A	Reference category	N/A
Creatinine 1.6 - 2 mg/dL	2.51 (2.23 – 2.83)	<.001	1.63 (1.42 - 1.88)	<.001
Creatinine >2 mg/dl	3.47 (3.18 - 3.80)	<.001	2.45 (2.17 - 2.76)	<.001

© 2021 Roth GA et al. *JAMA Network Open*.

Univariate association with mortality represents unadjusted odds ratios between each variable and inpatient mortality. Multivariate association represents adjusted odds ratios of our primary adjustment model reported in the paper.

eTable 4. Counts of Inpatients and Deaths, and In-Hospital Mortality Rate, by Month

	Inpatients, number	Deaths, number	In-hospital mortality rate, percent
March	5,039	1,077	21.4%
April	6,862	1,191	17.4%
May	2,574	312	12.1%
June	1,542	176	11.4%
July	1,549	175	11.3%
August	1,160	123	10.6%
September	739	95	12.9%
October	910	99	10.9%
November	361	23	6.4%

© 2021 Roth GA et al. *JAMA Network Open*.
